# Supplementary material for: How Does Selenium Intake Differ among Children (1–3 Years) on Vegetarian, Vegan, and Omnivorous Diets? Results of the VeChi Diet Study
Source: Nutrients. 2022 Dec 21;15(1):34. doi: 10.3390/nu15010034 (PMC9824336; doi:10.3390/nu15010034)
Supplement: Supplementary file 1 [file nutrients-15-00034-s001.zip › nutrients-2104983-supplementary.pdf]

Table S1: Adjusted mean daily selenium intake among 1- to 3-year-old children by dietary group.

				Basic model*		Final model	
	**VN** n = 139	**VG** n = 127	**OM** n = 164	*p*	Partial η^2	*p*	Partial η^2
**Adjusted mean selenium intake** (µg/d)[a]	2.78[1]	2.77	3.05[1]	0.062	0.013	< 0.001	0.058
**Adjusted mean selenium intake excluding Brazil nuts** (µg/d)[b]	17.51[1]	17.43	22.34[1]	<0.001	0.044	<0.001	0.047
**Adjusted mean selenium intake** (µg/1,000 kcal/d)[c]	2.85[1]	2.84	3.09[1]	0.105	0.011	<0.001	0.044

Vegan (VN); vegetarian (VG); omnivorous diet (OM). Values are given as means (x). * p-values and effect sizes were determined using ANCOVA, adjusting for age and sex. [a] In the fully adjusted model (log [x]), we adjusted for age, sex, weight-to-height Z-scores, season, socio-economic status, and energy intake (n = 430). [b] In the fully adjusted model, we adjusted for age, sex, weight-to-height Z-scores, season, socio-economic status, and energy intake (n = 423). [c] In the fully adjusted model (log[x]), we adjusted for age, sex, weight-to-height Z-scores, season, and socio-economic status (n = 423). [1] Values with the same superscripts are significantly different in the fully fitted model (p <0.001).
